# The Physician's Wife and the Things That Pertain to Her Life

**Published:** 1894-12

**Authors:** 


*The Physician's Wife and the Things that Pertain to her Life.* By ELLEN M. FIREBAUGH. Pp. xi., 186. Philadelphia: THE F. A. DAVIS COMPANY. London: F. J. REBMAN. 1894.

Of the making of books there is no end, but this one does not require much study and will not induce weariness. The doctor's wife admits the reader to her husband's smoking-room and entertains him with suitable discourse, such as many a country doctor's wife could give, though few could give it so well. The wily angler who remarks, "Huh! If you don't never do nothin' worse than fish on Sunday you 'll git to heaven, shore," and "Besides, you little goose, it ain't wicked to fish with a bent pin, because you 'll not ketch nuthin'," and the clover-huller who announces with a contemptuous snort, "I 've seed doctors before I ever seed *you;* and I 've done a heap o' docterin' myself, I 'll let ye know that! I 've buried three husbands and six children, an' I doctered 'em all myself," are followed by the excellent hint, "Doctor, when the weather is fine and the roads are good, do not wait for your wife to ask you now and then if you are going to the country to-day, and if it would be agreeable to you for her to accompany you. While you may answer 'Yes' with alacrity, it will still not be quite so pleasant to her as if the suggestion had come from you."

The following paragraph will come home to some of us: "When the doctor is sick he is capable, as we have seen, of growing despotic. Perhaps one reason for this may be that it is only a step from autocrat to despot; and when he is well he makes an excellent autocrat, as many physicians' wives have occasion to know. He is so accustomed to having his way and his say in the sick-room, which is right, that he wants to be the autocrat of the breakfast-table, the dinner-table, the tea-table, and the time between tables, which is not right."

We must refrain from further quotation. The above will suffice to show that the reader will be well entertained with humour, pathos, and good sense, infused with an agreeable flavour which we can only describe as American.

